# Cardiotoxicity in dual immune checkpoint inhibitors: a critical appraisal of signal interpretation and future directions

**DOI:** 10.1093/ehjcvp/pvag012

**Published:** 2026-03-19

**Authors:** Weikai Dong, Qifeng Song, Qiang Wang

**Affiliations:** Cardiovascular Medical Center, Nanjing Drum Tower Hospital, the Affiliated Hospital of Nanjing University Medical School, Nanjing, Jiangsu Province 210008, China; Faculty of Medicine, Yangzhou University, Yangzhou, Jiangsu Province 225009, China; Cardiovascular Medical Center, Nanjing Drum Tower Hospital, the Affiliated Hospital of Nanjing University Medical School, Nanjing, Jiangsu Province 210008, China

We have read with great interest the recent publication by Xia *et al*.,^1^ entitled ‘Cardiac Adverse Events Associated with Dual Immune Checkpoint Inhibitors: A Pharmacovigilance Analysis from the FDA Adverse Event Reporting System’.^1^ This study comprehensively evaluates the signals and also expounds on the onset time and clinical characteristics. This study provides valuable real evidence, but other methods and conceptual factors also need to be considered.

Firstly, the analysis does not first place dual Immune Checkpoint Inhibitor (ICI) cardiotoxicity in the context of other anticancer drug—induced cardiac dysfunction. VigiBase® large-scale study indicates that numerous oncological agents are correlated with cardiac dysfunction in a heterogeneous manner.^2^ Furthermore, disproportionality signals can be stronger than ICIs. Until standardized pharmacovigilance measures allow comparable benchmarking against other cardiotoxic anticancer drugs, it remains challenging to put the signals for dual ICI-related cardiotoxicity in clinical perspective and to gauge their relative specificity.

Secondly, large-scale assessments of the WHO pharmacovigilance database demonstrated that atrial fibrillation is a unique and independently enriched safety signal among anticancer therapies, distinct from other rhythm disturbances.^3^ The aggregate of the preferred terms may mask important clinical differences in the arrhythmic events. Hence, in a methodological frame, a structured subclassification of the cardiac signals is warranted to enforce accurate detection and mechanistic interpretation. This can include atrial fibrillation, ventricular arrhythmias, conduction disorders and sudden cardiac death.

Thirdly, this study did not involve prevention strategies for high-risk populations. Currently, expert consensus points out that cardiovascular risk assessment at baseline and structured monitoring strategies should be carried out for patients receiving cardiotoxic therapy,^4^ but routine use of drugs for ICI-related cardiotoxicity prevention is not recommended. More research is needed to better determine the optimal monitoring pathways for high-risk individuals.

Furthermore, cardiometabolic phenotyping was not included. According to detailed research studies of trastuzumab-mediated cardiotoxicity, integrated metabolic parameters, imaging, and functional studies facilitate the understanding of injury to the myocardium due to therapy.^5^ Using the same multidimensional phenotyping frameworks applied to ICI agents in the target population has the potential to unveil susceptible markers and monitoring algorithms early in illness.

In conclusion, the study provides important pharmacovigilance data regarding the cardiotoxicity of dual ICIs but must be followed on by future studies incorporating phenotypic characterization, mechanistic stratification of arrhythmias and evaluation of prevention strategies for precision cardio-oncology. Our advice is geared towards improving an already excellent article and we look forward to more insightful articles from the authors.

## Data Availability

No new data were generated or analysed in support of this research.
